# Advances in Non-Small Cell Lung Cancer Cellular Immunotherapy: A Progress in Dendritic Cell, T-Cell, and NK Cell Vaccines

**DOI:** 10.3390/cells14181453

**Published:** 2025-09-16

**Authors:** Mirza Masroor Ali Beg, Mohammad Aslam, Asma Ayaz, Muhammad Saeed Akhtar, Wajid Zaman

**Affiliations:** 1 Faculty of Medicine, Ala-Too International University, Bishkek 720048, Kyrgyzstan; mirzamasroor1@gmail.com; 2 Department of Chemical Engineering, Yeungnam University, Gyeongsan 38541, Republic of Korea; mohammadaslam13@yu.ac.kr; 3 Faculty of Sports Science, Ningbo University, Ningbo 315211, China; asma@nbu.edu.cn; 4 Department of Chemistry, Yeungnam University, Gyeongsan 38541, Republic of Korea; 5 Department of Life Sciences, Yeungnam University, Gyeongsan 38541, Republic of Korea

**Keywords:** non-small cell lung cancer, cell-based immunotherapy, dendritic cell, T cell, NK-cell vaccines, clinical outcomes, combination therapy, biomarkers, safety, adverse events

## Abstract

Over the past decade, cellular immunotherapy has emerged as a transformative strategy for non-small cell lung cancer (NSCLC), with dendritic-cell (DC) vaccines, T-cell vaccines, and natural killer (NK)-cell therapies demonstrating distinct mechanisms and clinical potential. DC vaccines capitalize on antigen presentation to prime tumor-specific T-cell responses, showing excellent safety profiles limited mainly to injection-site reactions and flu-like symptoms. While monotherapy has shown limited efficacy, combinations with checkpoint inhibitors or chemotherapy enhance immune activation and survival outcomes. Recent innovations, including neoantigen-loaded, mRNA-electroporated, and exosome-pulsed DCs, demonstrate improved immunogenicity and personalized approaches. T-cell vaccines, designed to activate cytotoxic CD8+ T-cell responses, have been tested across multiple platforms, including peptide-based (MAGE-A3), viral vector (TG4010/MUC1), and mRNA (CV9201/92) formulations. While the phase III MAGRIT trial presented no disease-free survival (DFS) benefit with adjuvant MAGE-A3 vaccination, the TG4010 vaccine improved progression-free survival (PFS; HR 0.66) and overall survival (OS; HR 0.67) in MUC1-positive NSCLC when combined with chemotherapy. Current strategies focus on personalized neoantigen vaccines and KRAS-targeted approaches (e.g., ELI-002), with ongoing phase III trials evaluating their potential in resectable NSCLC. NK-cell therapies have also shown promise, with early trials establishing the feasibility of autologous and allogeneic infusions, while engineered CAR-NK cells enhance tumor-specific targeting. Combination strategies with checkpoint inhibitors significantly improve response rates and PFS, revealing synergies between innate and adaptive immunity. Recent advances include cytokine-enhanced, memory-like NK cells to overcome immunosuppression and “off-the-shelf” products for broader clinical use. Together, these cellular immunotherapies represent a versatile and evolving frontier in NSCLC treatment, with ongoing research optimizing combinations, delivery platforms, and patient selection to maximize therapeutic benefit.

## 1. Introduction

Lung cancer remains the leading cause of cancer-associated mortality worldwide, accounting for approximately 1.8 million deaths each year [1]. Non-small cell lung cancer (NSCLC) constitutes about 85% of cases, and the 5-year survival rate for advanced disease is <20% [2]. Despite major advancements in lung cancer screening with low-dose CT and minimally invasive surgical procedures, a considerable proportion of patients with non-small cell lung cancer (NSCLC) are still discovered at an advanced stage. Although screening has increased the rate of early discovery in eligible individuals, overall, only about 25–30% of patients arrive with localized disease amenable to surgical resection to cure it [3]. Even after successful surgery, recurrence is common, and outcomes for advanced or metastatic NSCLC remain poor, particularly after the failure of first-line therapies [4,5].

Substantial advances in immuno-oncology have not eliminated relapse or primary resistance, prompting a shift toward more precise, individualized strategies, such as cellular immunotherapies [6]. These approaches involve ex vivo modification or expansion of immune effector cells, tumor-infiltrating lymphocytes (TILs), chimeric antigen receptor (CAR) T cells, T-cell receptor (TCR)-engineered T cells, cytokine-induced killer (CIK) cells, natural killer (NK) cells, and dendritic cells (DCs) to generate highly specific and durable antitumor responses [7,8].

Combination treatments are also gaining attention; co-administration of epidermal growth factor receptor tyrosine kinase inhibitors (EGFR-TKIs) with invariant NK T cells demonstrated acceptable safety and preliminary synergy in patients with EGFR-mutant NSCLC [9]. Nevertheless, solid tumors pose unique barriers, including an immunosuppressive microenvironment, physical impediments to lymphocyte infiltration, antigenic heterogeneity, and variable checkpoint expression. In addition, cellular products are constrained by complex manufacturing, high cost, and lengthy production timelines [10].

Despite these challenges, immunotherapy is redefining the therapeutic landscape for NSCLC. Unlike cytotoxic chemotherapy or radiotherapy, which indiscriminately damage healthy tissue, immunotherapy harnesses the patient’s immune system to selectively eradicate malignant cells, thereby reducing toxicity and potentially improving outcomes [11]. Emerging cellular modalities, such as TILs, CIK cells, DC vaccines, and genetically modified T cells are especially promising. TIL therapy, already successful in melanoma, has shown feasibility and safety in early NSCLC studies [12,13]. Similarly, adjuvant trials employing or lymph node-derived antigen-presenting cells report enhanced cytotoxic T-cell activation and encouraging survival signals [4].

Modern cancer immunotherapy now extends beyond checkpoint inhibitors and CAR-T cells to include bispecific T-cell engagers, neoantigen vaccines, and modulation of myeloid cells. Bispecific antibodies that concurrently bind T lymphocytes and tumor-associated antigens precisely redirect cytotoxicity toward malignant cells, increasing antitumor efficacy while limiting off-target toxicity [14]. It demonstrated that an mRNA neoantigen vaccination (RO7198457) generated strong T-cell responses in patients with metastatic melanoma, even after they were treated with PD-1 inhibitors. It proved that the vaccine could strengthen and prolong the body’s resistance to the cancer [15]. Moreover, reprogramming immunosuppressive candidates, including tumor-associated macrophages and myeloid-derived suppressor cells, can further augment T cell-mediated effects [16]. Together, these approaches represent a paradigm shift that addresses therapeutic resistance and enables highly individualized regimens. The present review summarizes the 10-year landscape of cell-based immunotherapies, mainly DCs, T cells, and NK cells for NSCLC, focusing on recent clinical advances, persisting challenges, and future directions for solid tumors.

## 2. Materials and Methods

This study combined data from ongoing and published clinical trials and studies assessing adoptive cellular treatments (ACTs) for NSCLC published between 2015 and 2025. We focused on three main therapeutic platforms: dendritic-cell (DC) vaccines, T-cell-based vaccines, and natural killer (NK)-cell therapies. The analysis considered both completed research and ongoing clinical trials to provide a comprehensive picture of recent developments.

We found relevant studies by carefully searching medical literature databases and clinical trial registries. The studies that are included must address at least one of three crucial elements: safety profiles, immunologic activity (such as DC cell, T cell, or NK cell responses), or clinical efficacy metrics like objective response rates (ORR), progression-free survival (PFS), and overall survival (OS).

## 3. Dendritic Cell (DC) Vaccines in NSCLC

**Figure 1:**
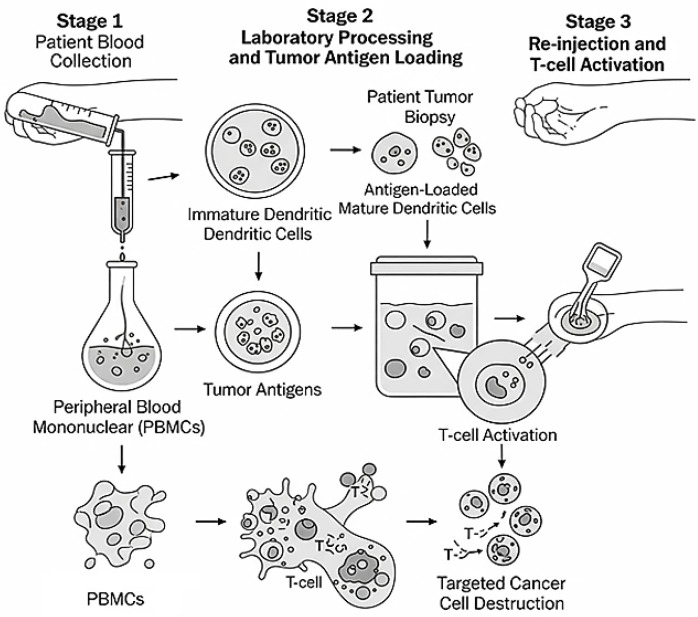
Diagram of dendritic-cell vaccine production and mechanism of action: From patient blood collection to tumor-specific T-cell activation.

A novel immunotherapeutic strategy for NSCLC is the use of DC vaccines, which capitalize on the DCs’ potent antigen-presenting capacity to provoke tumor-specific T-cell responses. DCs are expert antigen-presenting cells that can prime naïve T cells and elicit robust adaptive immune responses against antigens associated with cancer. The most common method for creating ex vivo DC vaccines for NSCLC is to separate patient autologous monocytes, turn them into DCs, and then load them with tumor-associated antigens (TAAs), like mucin 1 (MUC1), Wilms’ tumor 1 (WT1), or customized neoantigens. These antigen-loaded DCs are re-administered to the patient after maturing in order to trigger cytotoxic T lymphocyte (CTL) responses that specifically target cancerous cells (Figure 1).

The primary feature of dendritic cell (DC) vaccines’ favorable safety profile is their low-grade adverse events. Local injection site reactions and transient constitutional symptoms like fatigue and myalgia are the most common toxicities; dose-limiting toxicities have not been observed in more recent trials [17,18]. While monotherapy has achieved little tumor shrinkage, combination regimens—such as DC vaccines with chemotherapy, immune checkpoint inhibitors (anti-PD-1/PD-L1), or cytokine-induced killer cells—have shown greater immune activation and improved survival outcomes [19]. To enhance antigen presentation and combat tumor-induced immunosuppression, new strategies are being researched, such as exosome-pulsed DCs and mRNA-electroporated DCs [20]. Despite the lack of extensive phase III trials, DC vaccines may be most useful as maintenance therapy following chemoradiation or in cases with minimal residual disease. Ongoing research focuses on optimizing synergistic combinations, delivery strategies, and antigen selection to maximize therapeutic benefit [19,20].

Over the past decade, there has been a significant shift in the research on dendritic-cell (DC) vaccines for NSCLC. More recent efforts have clearly shifted toward customization, improved antigen delivery, and sensible combinations with systemic therapies, even though early-stage vaccines based on peptides and lysates established a good safety and immunogenicity baseline. Early trials conducted using the WT1- and MUC1-peptide methods [21,22,23] served to show that patients who had received intensive pretreatment could safely receive autologous monocyte-derived DCs, which consistently produce antigen-specific CD4+ and CD8+ responses. However, real tumor regressions were uncommon in these monotherapy settings, underscoring the limitations of single-antigen approaches and the suppressive characteristics of the NSCLC tumor microenvironment.

Randomized and single-arm combination experiments attempted to use chemotherapy-induced antigen release and immunogenic cell death in the hope that concurrent DC vaccination could benefit from increased antigen availability. Trial design heterogeneity, small sample sizes, and inconsistent endpoint reporting complicate definitive efficacy claims, even though some analyses show modest improvements in PFS in particular subgroups, and interim data demonstrate acceptable tolerability and enhancement of peripheral immune markers [24].

A fundamental conceptual shift was brought in that neoantigen-directed DC vaccinations (ongoing neoantigen trials) are feasible within clinical timescales and may increase the availability of private neoantigen-specific T cell clones, a crucial development for personalization. Neoantigen vaccines address tumor heterogeneity by targeting mutational epitopes specific to each patient. They have repeatedly shown strong immunogenicity with occasional partial responses, suggesting that tailored antigen selection can have more profound biological effects than tumor-associated antigens that are sold commercially [25]. Concurrently, the field prioritized combination with immune checkpoint blockade: Early combination arms report enhanced systemic activation and increased intratumoural T-cell infiltration when DC vaccination is administered prior to or concurrently with anti-PD-1 therapy [26]. While mRNA electroporation strategies [27] and exosome-pulsed antigen delivery [28] present technological advancements aiming for scalable, multi-epitope presentation and more consistent antigen expression, hybrid cell therapies [29] combine DC priming with cytotoxic cellular effectors to amplify immediate tumoricidal activity.

The majority of adverse effects are mild injection site reactions, transient fever, or fatigue; all vaccines are safe. Reproducible immunogenicity is indicated by the formation of T cell receptor clonotypes, IFN-γ ELISPOTs, multimer staining, and, in some cases, an increase in tumor-infiltrating lymphocytes. Clinical efficacy as monotherapy is still low in advanced, heavily pretreated populations, although the most compelling indications are in combination treatments, earlier adjuvant settings, or when vaccines are truly customized. Some of the methodological issues that require attention include small and heterogeneous patient populations, different results (immune vs. clinical), limited randomized comparisons, and variations in DC manufacturing (source cells, maturation cocktails, antigen-loading procedure). Furthermore, to turn frequent immunologic responses into steady, long-lasting clinical effects, adjuvant immune modulators may need to be integrated with multi-platform antigen delivery (neoantigen peptides, mRNA, exosomes). Overall, DC vaccines have been proven to be safe and physiologically active in NSCLC during the 2015–2025 decade, and current tendencies toward combination and personalization techniques offer a viable way to improve patient outcomes, pending the outcomes of larger, ongoing, well-controlled trials (Table 1).

## 4. T-Cell-Based Vaccines in NSCLC

**Figure 2:**
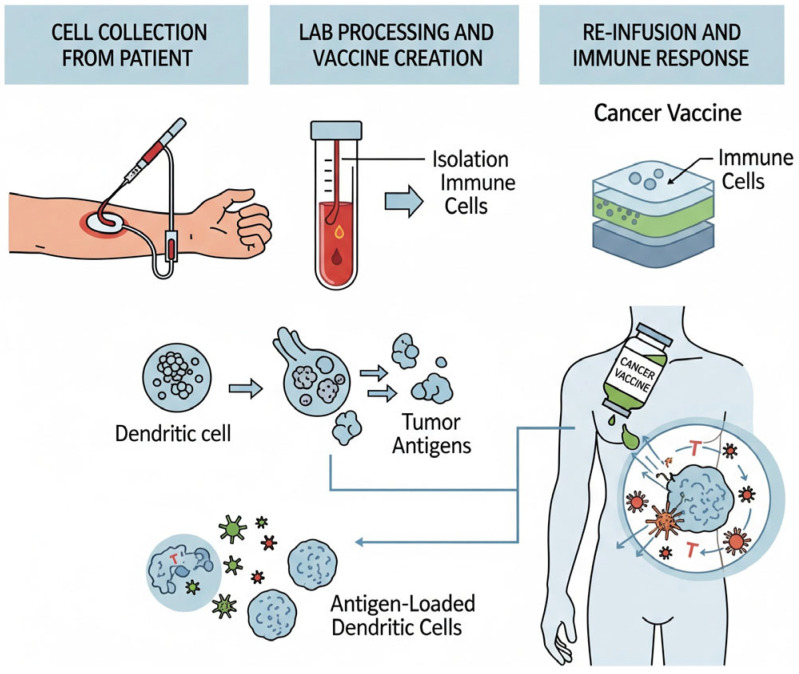
Demonstration of T-cell vaccine preparation and administration in NSCLC, and illustration of the treatment approach.

NSCLC is treated with T-cell vaccinations, a form of targeted immunotherapy that stimulates the patient’s adaptive immune system to distinguish and eradicate tumor cells (Figure 2). By exposing the immune cells to tumor-associated antigens (TAAs) or patient-specific neoantigens, these vaccines primarily work by inducing cytotoxic CD8+ T lymphocyte (CTL) responses, which have the ability to kill cancer cells [32,33] specifically. The mechanism of action involves presenting antigens through Major Histocompatibility Complex (MHC) molecules, which prime and multiply T lymphocytes specific to antigens [34]. Clinical trials in early phase have demonstrated the safety and immunogenicity of T-cell vaccines in NSCLC. However, their clinical efficacy has been limited, likely due to tumor heterogeneity, immune checkpoint-mediated suppression, and the immunosuppressive tumor microenvironment [35,36].

Between 2015 and 2025, a variety of T-cell-based vaccines, such as peptide, viral vector, and mRNA vaccine platforms, were tested in NSCLC. These tactics aimed to enhance tumor-specific T-cell responses by employing tailored neoantigens or tumor-associated antigens (TAAs). The results of completed and ongoing trials indicate that the efficacy profile is variable, with immune activation being more consistent than survival benefits.

One of the earliest and most well-known studies was TIME, which assessed TG4010, a modified vaccinia Ankara (MVA) viral vector that contained the tumor-associated antigen MUC1 and interleukin-2 (IL-2). TG4010 and first-line chemotherapy significantly improved overall survival (OS) (HR 0.67, *p* = 0.018) and progression-free survival (PFS) (hazard ratio [HR] 0.66, *p* = 0.010) in the predefined TrPAL-low subgroup of patients with MUC1-positive advanced NSCLC. Importantly, immunomonitoring revealed signs of epitope spreading, suggesting that a broader antitumor immune response was triggered. The vaccine was generally well tolerated, and there were no unexpected side effects [37].

The START study evaluated a peptide vaccine that targets the melanoma-associated antigen A3 (MAGE-A3) in combination with treatment for stage IIIB/IV NSCLC. When compared to chemotherapy alone, the vaccination did not produce a statistically significant improvement in PFS or OS, despite being immunogenic [38]. MAGE-A3 peptide immunization was investigated in the MAGRIT phase III trial in stage IB–IIIA NSCLC patients who had undergone adjuvant full resection. No disease-free survival (DFS) benefit over a placebo was found. The program was later discontinued for NSCLC, despite the fact that the antigen target was still of interest for other cancers. The tolerance of T cell-based vaccination strategies was once again shown by positive safety results [39]. Individualized neoantigen vaccination became more popular in later years. Du et al. studied the effects of a customized peptide vaccine, with or without chemotherapy, on patients with advanced NSCLC who had received standard treatment. The tailored formulation generated robust CD8 T-cell responses to patient-specific neoantigens. Several patients in the clinical context experienced stable illness, supporting the idea of tailored antigen selection, even though no discernible improvement in survival was observed in the small phase I setting [40]. An mRNA-based vaccine, CureVac’s CV9201/92 mRNA vaccination program, targeted multiple tumor antigens and was tested both in isolation and in combination with PD-L1 inhibition. The combination strategy showed encouraging immune activation and disease control in subgroups of patients with advanced NSCLC, suggesting a synergistic effect with checkpoint inhibition. Safety was judged adequate despite the lack of known dose-limiting toxicities [41].

In recent years, there has been an expansion into customized mRNA vaccination strategies. The current INTerpath-009 (mRNA-4157/V940) trial by Moderna and Merck is evaluating a patient-specific mRNA neoantigen vaccination plus pembrolizumab in the adjuvant context for resected high-risk NSCLC. This global phase III trial is recruiting patients who did not show a pathological complete response after neoadjuvant therapy, with the main objective being DFS; however, the results are expected after 2025 [42].

The ELI-002 (AMPLIFY) initiative uses commercially available KRAS mutant peptides (G12/G13) and a lymph node-targeted amphiphile peptide vaccination platform. Expansion into NSCLC cohorts was prompted by early phase I findings showing substantial immunogenicity in minimum residual disease groups. The platform’s architecture improves the delivery of antigen to lymphoid tissue, which may increase the priming effectiveness of CD8 T cells [43].

Personalized neoantigen mRNA vaccines have been assessed in advanced non-small cell lung cancer in several early-phase trials. The majority of patients experience detectable neoantigen-specific T-cell responses, and manufacturing viability has been validated across trials. Combinations with immune checkpoint inhibitors (ICIs) seem to increase immunogenicity and clinical activity, and safety profiles have been positive [44]. Finally, a customized multi-peptide vaccine has been tested in combination with ICIs against a range of solid cancers, including non-small cell lung cancer, as part of Mount Sinai’s PGV001 program [45]. Preliminary findings indicate early disease control signals, strong immune activation, and feasibility in NSCLC subgroups (Table 2).

## 5. Natural Killer (NK)-Cell-Based Vaccines in NSCLC

**Figure 3:**
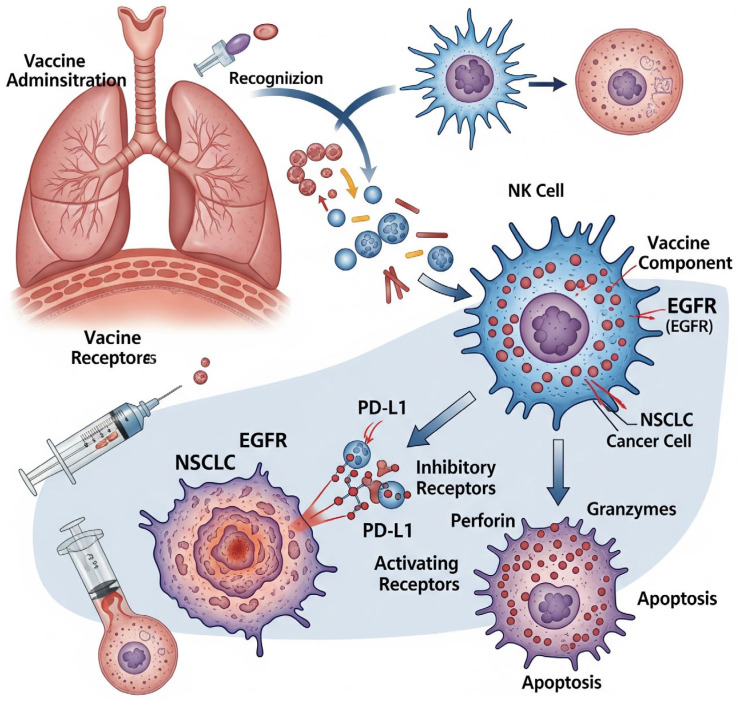
Demonstration of the NK-cell vaccine mechanism in NSCLC, and illustration of the treatment approach.

Natural killer (NK)-cell-based vaccines and treatments are examples of new immunotherapeutic approaches for NSCLC. By using death receptor signaling, perforin/granzyme release, and antibody-dependent cellular cytotoxicity (ADCC), NK cells, innate lymphocytes, can eliminate tumor cells without first sensitizing the cells [46]. In NSCLC, inhibitory checkpoints are upregulated and activating ligands are downregulated, necessitating the therapeutic enhancement of NK activity [47] (Figure 3).

From 2015 to 2025, clinical research has steadily increased our understanding of NK-cell-based therapies for NSCLC, highlighting the therapeutic potential and safety in a variety of settings and patient populations.

A phase I trial involved autologous NK cell adoptive transfer and IL-2 in patients with advanced NSCLC. The treatment was well tolerated, with few side effects, and some patients showed signs of immune activation along with partial tumor reduction, suggesting that NK cell treatments could be used safely in this population [48]. It has been investigated that the use of ex vivo-generated NK cells and chemotherapy in patients with refractory or relapsed NSCLC [49]. The Phase I clinical study demonstrated increased cytotoxicity of NK cells without significant harm, confirming the feasibility of integrating NK cell-based strategies into existing treatment plans [49]. These outcomes were supported by a study by Choi et al. in patients with advanced NSCLC who had undergone intensive pretreatment. The safety of autologous NK cells that were expanded and administered with IL-2 supported the potential use of NK cells as an adjuvant therapy. These cells also contributed to disease stability in some patients [50]. It has been demonstrated the safety and efficacy of allogeneic NK cell treatment for stage III/IV non-small cell lung cancer in a Phase I trial. This increased their practical relevance by implying a major step toward “off-the-shelf” treatments that could be produced and used without requiring patient-specific cells [51]. In advanced non-small cell lung cancer, a Phase I study of engineered NK cells ± atezolizumab revealed that the treatment was safe, well-tolerated, and showed early anti-tumor activity [52].

A Phase I/II study combining NK-cell therapy with pembrolizumab in advanced metastatic NSCLC demonstrated that the treatment was safe and well tolerated, enhanced anti-tumor activity, and improved objective response rate (ORR) and progression-free survival (PFS) [53]. NK cell vaccinations administered in combination with PD-1 blockade produced considerably better overall response rates and progression-free survival compared to checkpoint inhibition alone. As per these results, innate and adaptive immune activation may cooperate to enhance anticancer efficacy [54]. Current trials, such as NCT03941457 (2021) and NCT04551885 (2023), are actively evaluating the safety and efficacy of NK cell infusion in combination with immune checkpoint inhibitors or chemotherapy for metastatic non-small cell lung cancer. Immune system activation and good tolerability are suggested by preliminary data [55,56].

There are significant safety challenges in developing cancer vaccines, particularly in selecting TAAs. Many TAAs have similar regions, called shared epitopes, that are also present in normal human proteins, even though they are commonly selected because they are primarily found in cancer cells. Because common sequences in antigens linked to tumors may cause autoimmune reactions by inadvertently targeting healthy tissues, cancer vaccines pose safety risks. This risk is greater for tissue-specific antigens like ASCL2, KLK2, TPTE, CLDN6, and PSMA, which may evade early detection in initial testing or become more severe when taken with checkpoint inhibitors. By activating regulatory T cells, these shared sequences may reduce efficacy, even when there are no obvious autoimmune reactions. Therefore, developing safer and more effective vaccines in the future requires careful target sequence selection and sophisticated antigen design [57] (Table 3).

## 6. Conclusions

Over the past ten years, cellular immunotherapies, such as T-cell, NK-cell, and dendritic-cell (DC) vaccines, have emerged as promising treatments for NSCLC. DC vaccinations can boost the immune system and are safe, but they work best when combined with other therapies. T-cell vaccines, particularly those that employ specific neoantigen techniques, have been shown to elicit potent anti-tumor immune responses. NK cell treatments have advanced from straightforward infusions to intricate customized forms with improved efficacy and durability. Although these treatments are generally well tolerated, there are still challenges in overcoming the tumor’s immune system resistance and ensuring consistent responses in each patient. The most successful strategies combine these cellular therapies with drugs that have already received approval, like checkpoint inhibitors or chemotherapy. Future research should focus on improving patient selection using biomarkers, standardizing production processes, and refining these combinations to make these innovative medications accessible to more NSCLC patients.

## Figures and Tables

**Table 1:** Summary of completed and ongoing clinical trials of dendritic-cell (DC) vaccines in NSCLC, highlighting safety, immunogenicity, and efficacy outcomes.

Year	Phase & Design	Intervention	Population	Key Outcomes	Reference
2017	Phase I, pilot study	Autologous DCs pulsed with WT1 peptide + chemotherapy	Advanced NSCLC	Combination therapy was safe and feasible. WT1-specific immune responses were observed and correlated with a trend towards improved survival	[21]
2018	Phase II, randomized	Allogeneic DCs/CIK cells + Chemotherapy vs. Chemotherapy alone	Stage IIIB/IV NSCLC	Improved ORR and DCR. Significantly longer PFS (6.5 vs. 4.3 mo) and OS (21.0 vs. 14.5 mo) in the combination group.	[24]
2017	Phase I/II, open-label	DCs with MUC1 peptide	Advanced NSCLC, refractory cases	Safe, PR 2/16, SD 7/16, increased MUC1-specific T-cell responses	[22]
2018	Phase I, open-label	DCs with WT1 + MUC1 peptides	Post-first-line chemotherapy	Safe, enhanced antigen-specific CTL activity, median PFS ~4.5 months	[23]
2020	Phase I and II, single arm	DCVAC/LuCa (allogeneic dendritic cell vaccine) + Carboplatin/Pemetrexed chemotherapy	Advanced NSCLC	Promising median Overall Survival (OS) of 15.5 months. Disease Control Rate (DCR) of 70.6%. Associated with increased cytotoxic T-cell and NK cell activity.	[25]
2020	Phase II, randomized	DCs + chemotherapy	Stage III NSCLC post-CRT	Ongoing, interim safety confirmed, PFS results awaited	[30]
2021	Phase I/II, open-label	Autologous DCs pulsed with tumor lysate + Nivolumab	Advanced NSCLC	Well-tolerated and induced neoantigen-specific T-cell activation, culminating in a durable complete response, demonstrating preliminary efficacy	[31]
2016	Phase I, open-label	Autologous DCs electroporated with mRNA encoding personalized neoantigens	Advanced NSCLC	Well-tolerated, robust mutation-specific T-cell responses in in a patient with PD-1 blockade-resistant NSCLC	[28]
2015	Phase II, multi-centre	Allogeneic DC/CIK + Anti-PD-1	Advanced NSCLC post-IO failure	Enhanced ORR and DCR; prolonged PFS; well-tolerated.	[29]
20125	Phase I/II, open-label	DCs with exosome pulsed tumor antigens	Advanced NSCLC	Ongoing, early immune response data are promising	[27]

**Table 2:** T-cell-based vaccines in NSCLC (2015–2025) show a decade of clinical trials, key outcomes, and future directions.

Year	Phase & Design	Intervention	Population	Key Outcomes	Reference
2015	Phase IIb, randomized	Viral-vector (MVA) vaccine encoding MUC1 + IL-2	Advanced MUC1-positive NSCLC, first line	In the TrPAL-low subgroup: improved PFS (HR 0.66) and OS (HR 0.67), epitope spreading, tolerable safety	[37]
2012	Phase II, randomized	Peptide vaccine + chemotherapy	Stage IIIB/IV NSCLC	Immunogenic but no significant PFS/OS benefit, acceptable safety	[38]
2017	Phase III, randomized (adjuvant)	Adjuvant peptide vaccine	Completely resected NSCLC (IB-IIIA)	No DFS benefit, safe, program halted for NSCLC	[39]
2019	Phase I, open-label	Personalized peptide neoantigen vaccine ± chemo	Advanced NSCLC post-chemo	Induced neoantigen-specific CD8^+^ T cells, stable disease in a subset	[40]
2022	Phase I/II, open-label	mRNA multivalent vaccine + PD-L1 blockade	Advanced NSCLC	Safe, immune activation, disease control in subsets with ICIs	[41]
2024	Phase III, randomized (adjuvant)	Personalized mRNA neoantigen vaccine + pembrolizumab	Resected high-risk NSCLC	Recruiting; primary endpoint DFS, results pending	[42]
2023	Phase I	Lymph node-targeted amphiphile peptide vaccine	KRAS-mutant solid tumors; lung planned	High immunogenicity in MRD cohorts; expansion to lung planned, favorable safety	[43]
2023–2025	Phase I, single-arm	Personalized neoantigen mRNA vaccines	Advanced NSCLC	Feasible manufacturing; strong immunogenicity, safe, clinical activity variable	[44]
2024–2025	Phase I	Personalized multi-peptide vaccine + ICI	Mixed solid tumors including NSCLC	Feasible, immunogenic, early disease control signals	[45]

**Table 3:** The clinical trial environment for natural killer (NK) cell immunotherapy in NSCLC (2015–2025), with safety, efficacy, and emerging patterns.

Year	Phase & Design	Intervention	Population	Key Outcomes	Reference
2018	Preclinical	PD-1/PD-L1 immune checkpoint blockade with focus on NK cell contribution	Advanced NSCLC	efficacy; their loss reduced benefit, highlighting NK cell–intrinsic PD-1/PD-L1 signaling in tumor immunity	[48]
2019	Phase I	Ex vivo-expanded NK cells + cytotoxic T lymphocytes	NSCLC, relapsed or refractory	Good safety, enhanced NK cell cytotoxicity	[49]
2024	Phase I/IIb, open-label	Autologous NK cells + cytotoxic chemotherapy ± cetuximab	NSCLC, heavily pretreated	Safe, no dose-limiting toxicities; 25% objective response rate, 100% disease control, median PFS 143 days	[50]
2010	Phase I	Allogeneic NK-cell therapy	Advanced NSCLC	Feasibility and safety demonstrated	[51]
2023	Phase I	Engineered NK cells) ± Atezolizumab	Advanced NSCLC patients	Safety, tolerability, preliminary efficacy	[52]
2020	Phase I/II	NK-cell + Pembrolizumab	Advanced Metastatic NSCLC	Safe and well-tolerated; enhanced anti-tumor activity; improved ORR and PFS	[53]
2021	Phase II, randomized	NK-cell vaccine + anti-PD-1 therapy	Advanced NSCLC	Improved PFS vs. control, manageable toxicity	[54]
2020	Phase I/II	NK cell infusion + chemotherapy	Advanced NSCLC	Ongoing, safety data pending	[55]
2020	Phase II	Allogeneic NK-cell vaccine + chemotherapy	Metastatic NSCLC	Ongoing	[56]

## Data Availability

This published paper contains all of the information generated or analyzed during this research. However, the reader can ask the corresponding author for any additional data that were used or examined in this study on reasonable request.
